# Solubilization and Controlled Release Strategy of Poorly Water-Soluble Drugs

**DOI:** 10.3390/ph15111353

**Published:** 2022-11-02

**Authors:** M. A. Peña

**Affiliations:** Unidad Docente de Farmacia y Tecnología Farmacéutica, Facultad de Farmacia, Universidad de Alcalá, Alcalá de Henares, E-28871 Madrid, Spain; angeles.pena@uah.es; Tel.: +34-918854725

The processes of solubilization and controlled release of drugs that are poorly soluble in water are highly relevant in drug preformulation studies in pharmaceutical development.

In recent years, a large number of drugs have been designed and marketed, but almost 70% of them have low water solubility, which limits their dissolution rate, and consequently their bioavailability. Undoubtedly, this is a problem with repercussions for the pharmaceutical industry, so the discovery of new technological tools for the hydrosolubilization of drugs that do not affect their physical/chemical and therapeutic properties will constitute an extraordinary achievement.

On the other hand, from a pharmacological point of view, the search for knowledge of the interaction mechanism and the different properties of controlled drug release systems is of great importance in drug formulation, in order to achieve the release and absorption of the appropriate amount of the drug and at the precise time and place.

Considering all of the above, this Special Issue has paid special attention to the latest advances in the solubilization and controlled release strategy of drugs that are poorly soluble in water. Authors were invited to submit original research or review articles on these topics, including the development of novel solubilization strategies, and innovative methods of obtaining new controlled-release pharmaceutical forms. A total of 21 articles were submitted for publication, and those accepted (47.6% acceptance rate) can be found online; this Special Issue comprises 10 of those original research papers.

The properties of the carrier are important for the elaboration of solid dispersions; polymers such as polyvinylpyrrolidone (PVP), hydroxypropylmethylcellulose (HPMC), polymethacrylates, croscarmellose sodium, cyclodextrins or maltodextrins can improve the wetting of the drug and the pharmacological efficacy of the drugs. One of the ways to improve the bioavailability of drugs is through the use of solid dispersions developed using any of these novel carriers [1], or by converting the drug to amorphous forms using the freeze-drying technique and the help of certain excipients [2]. The applied amorphization technique has many advantages in relation to the possibility of upscaling this process with good performance. In this study, Wiergowska et al. (2021) used HPMC and β-cyclodextrin (β-CD) as effective modifiers of solubility and permeability for vardenafil hydrochloride, suggesting the possibility of achieving a stronger pharmacological effect. Currently, amorphization is considered as one of the best methods to administer insoluble drugs, especially those that belong to groups II and IV of the biopharmaceutical classification system (BCS) [3]. With both techniques of hydrosolubilization, the increase in solubility was related to the decrease in crystallinity, due to the inclusion of drug molecules within the carrier chains.

It is very interesting to note the appearance of new modern excipients, such as chitosan, which is capable of improving the biocompatibility properties and increasing the solubility of poorly water-soluble drugs. A large number of researchers currently show results with great potential in the use of excipients in modern oral or topical drug delivery systems [4]. Hydrophilic polymers and monomers have been widely used to improve the solubilization of poorly soluble drugs, showing excellent results against hydrophobic polymers, polyethylene glycols, β-CD, chondroitin sulfate, poloxamers, alginate sodium or PVP [5].

Secondly, lipid-based drug delivery systems consist of a very diverse group of formulations, in which each one has variable functional and structural properties, meaning that they are susceptible to being modified when the composition of the drugs varies. Lipid excipients and other additives facilitate the bioavailability of drugs that are poorly soluble in water. Delivery systems such as nanoemulsions, solid lipid nanoparticles (SLN), nanostructured lipid carriers (NLC), and self-emulsifying drug delivery systems (SEDDS) constitute an excellent example of systems that achieve drug delivery with low water solubility and other associated advantages. They can protect loaded drugs from chemical and enzymatic degradation, and gradually release drugs from the lipid matrix into the blood, resulting in improved therapeutic profiles compared to free drugs; as in the case of nanosuspensions and nanocrystals, they can achieve great suitability in scaling procedures, with a minimum amount of excipients [6,7,8]. For this reason, in pharmaceutical development, nanotechnology is essential to improve the oral bioavailability of drugs in the treatment of many pathologies, especially nanogels/nanomatrices, which are state-of-the-art drug delivery systems.

Bilosomes constitute a promising biocompatible nanocarrier for modulating the oral administration of drugs such as lycopene, a drug with anticancer, antioxidant, cardioprotective, neuroprotective, anti-inflammatory, antiplatelet, and antihypertensive effects [9]. Bilosomes, vesicles that incorporate bile salts, are emerging as colloidal carriers to improve the oral delivery of encapsulated drugs. In addition, bile salts can act as intestinal permeation enhancers, triggering increased oral bioavailability of the drug after bisomal encapsulation [10].

Finally, autonanoemulsifying drug delivery systems (SNEDDS), which are multicomponent systems that contain a lipophilic drug in a mixture of an oil or a lipid and a surfactant, or a mixture of surfactants and also a co-solvent, represent an interesting option to improve the limited water solubility of drugs. When in contact with gastrointestinal fluids and under the influence of digestive and gastrointestinal motility, SNEDDS spontaneously form clear nanoemulsions in which the formulated drug is solubilized.

Formulations known as SNEDDS are usually liquid formulations, which are also known as liquid-SNEDDS (L-SNEDDS). L-SNEDDS allow for high loadings with poorly water-soluble drugs and can be prepared fairly quickly and by simple means. However, the fact that they are liquid formulations also has some disadvantages. Various manufacturing technologies, such as adsorption by a solid carrier, wet granulation, spray drying, lyophilization and supercritical fluid processes, can be used to convert L-SNEDDS to solid SNEDDS (S-SNEDDS). Currently, the most common formulation approach currently used to produce S-SNEDDS is adsorption of L-SNEDDS by a solid carrier or via hot melt extrusion (HME) [11,12].

In conclusion, this Special Issue has highlighted the advances and research in the field of pharmaceutical and biopharmaceutical technology to address and solve the problems of poorly water-soluble drugs, to enhance dissolution rates and permeability. It is important to highpoint that the following methods have been explored: the use of solid dispersions, such as the one obtained with maltodextrin as carrier agent; procurement amorphous drugs by lyophilisation with saccharides; chitosan as a valuable carrier for oral and topical delivery systems; nanocarrier-based gel for topical delivery with chitosan; the use of nanocrystals to design tabs in cap systems; bilosomes as nanoplatforms for oral delivery and modulated in vivo antimicrobial activity or preparation of S-SNEDDS by co-extrusion of liquid SNEDDS and polymeric carriers.

## References

[1] Benavent C., Torrado-Salmerón C., Torrado-Santiago S. (2021). Development of a solid dispersion of nystatin with maltodextrin as a carrier agent: Improvements in antifungal efficacy against *Candida* spp. Biofilm infections. Pharmaceuticals.

[2] Wiergowska G., Ludowicz D., Wdowiak K., Miklaszewski A., Lewandowska K., Cielecka-Piontek J. (2021). Combinations of freeze-dried amorphous vardenafil hydrochloride with saccharides as a way to enhance dissolution rate and permeability. Pharmaceuticals.

[3] Deshmukh A.S., Tiwari K.J., Mahajan V.R. (2017). Solubility enhancement techniques for poorly water-soluble drugs. Int. J. Pharm. Sci. Nanotechnol..

[4] Sip S., Paczkowska-Walendowska M., Rosiak N., Miklaszewski A., Grabańska-Martyńska K., Samarzewska K., Cielecka-Piontek J. (2021). Chitosan as valuable excipient for oral and topical carvedilol delivery systems. Pharmaceuticals.

[5] Łyszczarz E., Hofmanová J., Szafraniec-Szczęsny J., Jachowicz R. (2020). Orodispersible films containing ball milled aripiprazole-poloxamer^®^ 407 solid dispersions. Int. J. Pharm..

[6] Noreen S., Pervaiz F., Ashames A., Buabeid M., Fahelelbom K., Shoukat H., Maqbool I., Murtaza G. (2021). Optimization of Novel Naproxen-Loaded Chitosan/Carrageenan Nanocarrier-Based Gel for Topical Delivery: Ex Vivo, Histopathological, and In Vivo Evaluation. Pharmaceuticals.

[7] Sreeharsha N., Naveen N.R., Anitha P., Goudanavar P.S., Ramkanth S., Fattepur S., Telsang M., Habeebuddin M., Answer M.K. (2022). Development of nanocrystal compressed minitablets for chronotherapeutic drug Delivery. Pharmaceuticals.

[8] Tashan E., Karakucuk A., Celebi N. (2019). Optimization and in vitro evaluation of ziprasidone nanosuspensions produced by a top-down approach. J. Drug Deliv. Sci. Technol..

[9] Binsuwaidan R., Sultan A.A., Negm W.A., Attallah N.G.M., Alqahtani M.J., Hussein I.A., Shaldam M.A., El-Sherbeni S.A., Elekhnawy E. (2022). Bilosomes as nanoplatform for oral delivery and modulated in vivo antimicrobial activity of lycopene. Pharmaceuticals.

[10] Pavlović N., Goločorbin-Kon S., Ðanić M., Stanimirov B., Al-Salami H., Stankov K., Mikov M. (2018). Bile acids and their derivatives as potential modifiers of drug release and pharmacokinetic profiles. Front. Pharmacol..

[11] Dokania S., Joshi A.K. (2015). Self-microemulsifying drug delivery system (SMEDDS)—Challenges and road ahead. Drug Deliv..

[12] Schmied F.-P., Bernhardt A., Klein S. (2022). Preparation of solid self-nanoemulsifying drug delivery systems (S-SNEDDS) by co-extrusion of liquid SNEDDS and polymeric carriers—A new and promising formulation approach to improve the solubility of poorly water-soluble drugs. Pharmaceuticals.

